# Gene expression changes in spinal motoneurons of the SOD1^G93A^ transgenic model for ALS after treatment with G-CSF

**DOI:** 10.3389/fncel.2014.00464

**Published:** 2015-01-20

**Authors:** Alexandre Henriques, Stefan Kastner, Eva Chatzikonstantinou, Claudia Pitzer, Christian Plaas, Friederike Kirsch, Oliver Wafzig, Carola Krüger, Robert Spoelgen, Jose-Luis Gonzalez De Aguilar, Norbert Gretz, Armin Schneider

**Affiliations:** ^1^INSERM, U1118, Mécanismes Centraux et Péripheriques de la NeurodégénérescenceStrasbourg, France; ^2^UMRS1118, Fédération de Médecine Translationnelle de StrasbourgUniversité de Strasbourg, France; ^3^Sygnis Bioscience GmbH & Co KGHeidelberg, Germany; ^4^Medical Research Center, Medical Faculty Mannheim, University of HeidelbergMannheim, Germany

**Keywords:** motoneuron, ALS, G-CSF, neurodegeneration, mouse model, gene expression, laser microdissection

## Abstract

**Background:** Amyotrophic lateral sclerosis (ALS) is an incurable fatal motoneuron disease with a lifetime risk of approximately 1:400. It is characterized by progressive weakness, muscle wasting, and death ensuing 3–5 years after diagnosis. Granulocyte-colony stimulating factor (G-CSF) is a drug candidate for ALS, with evidence for efficacy from animal studies and interesting data from pilot clinical trials. To gain insight into the disease mechanisms and mode of action of G-CSF, we performed gene expression profiling on isolated lumbar motoneurons from SOD1^G93A^ mice, the most frequently studied animal model for ALS, with and without G-CSF treatment.

**Results**: Motoneurons from SOD1^G93A^ mice present a distinct gene expression profile in comparison to controls already at an early disease stage (11 weeks of age), when treatment was initiated. The degree of deregulation increases at a time where motor symptoms are obvious (15 weeks of age). Upon G-CSF treatment, transcriptomic deregulations of SOD1^G93A^ motoneurons were notably restored. Discriminant analysis revealed that SOD1 mice treated with G-CSF has a transcriptom close to presymptomatic SOD1 mice or wild type mice. Some interesting genes modulated by G-CSF treatment relate to neuromuscular function such as CCR4-NOT or Prss12.

**Conclusions**: Our data suggest that G-CSF is able to re-adjust gene expression in symptomatic SOD1^G93A^ motoneurons. This provides further arguments for G-CSF as a promising drug candidate for ALS.

## Introduction

Amyotrophic lateral sclerosis (ALS) is a devastating neurodegenerative disease that results in progressive loss of motoneurons, motor weakness, and death within 1–5 years after disease onset. Although more than 130 years have passed since the disease was first described by Charcot its etiology is still enigmatic, precluding a truly rational drug development. Motoneurons in the spinal cord and the motor unit are the common endpoint in all hypotheses concerning pathophysiology (Gordon, 2013; Turner et al., 2013). The transgenic SOD-1^G93A^ mice are a model for ALS and recapitulate most of the ALS pathology, including loss of motor units. The first evidence of stress on the motor axis in this model occurs early before appearance of motor deficit (Frey et al., 2000).

G-CSF was primarily described as a hematopoietic factor able to stimulate the proliferation and differentiation of myeloid precursors. It is used for the treatment of chemotherapy-induced neutropenia and for bone marrow transplants (Kobbe et al., 2009; Yang et al., 2012). G-CSF has more recently been uncovered as a neurotrophic factor able to rescue motoneurons from apoptosis, protect motor functions and enhance overall survival of SOD1^G93A^ mice through the preservation of motor units (Pitzer et al., 2008; Henriques et al., 2010a,b, 2011).

Pilot clinical studies have investigated the potential of G-CSF as a drug candidate in ALS. Administrations were mainly subcutaneous with limited duration (unique or repeated injection for up to 5 days) and enrollment ranged from 10 to 39 patients. Adverse effects were minor and the drug was well tolerated in all studies. Effects of G-CSF on disease progression are unclear since all studies suffer from small cohort sizes. One study concluded that G-CSF treatment did not bring benefit for patients (Nefussy et al., 2010), while others either reported slower disease progression upon treatment (Martinez et al., 2009; Zhang et al., 2009), reduced damage of white matter tracts (Duning et al., 2011) or lower level of pro-inflammatory markers in the cerebrospinal fluid (Chio et al., 2011).

Effects of G-CSF treatment in SOD-1^G93A^ mice have been examined at the clinical, electrophysiological, and histological level. Here we sought to determine if and what changes on the gene expression level in alpha motoneurons would be related to G-CSF treatment. Therefore, our objective was to (1) define changes on the mRNA level that occur in spinal motoneurons in an animal model of ALS, the SOD1^G93A^ mice and (2) characterize the effects of G-CSF on detected changes in the transcriptome of those motoneurons. We combined laser microdissection (LMD), RNA amplification, and gene expression profiling on DNA arrays to study these cell-type specific expression changes.

## Materials and methods

### ALS model

We used mice transgenic for the SOD1^G93A^ mutation (Gurney et al., 1994) on a C57/bl6 background (B6.Cg-Tg(SOD1-G93A)1Gur/J strain; Jackson laboratory, Bar Harbor, Maine, USA), harboring the high copy number of the mutant allele human SOD1. The hemizygous line was maintained by mating transgenic males with C57BL/6 wild-type females. Transgenic littermate females were used in all experiments. They were either treated with vehicle (250 mM sorbitol, 0.004% Tween-80 and 10 mM sodium acetate buffer) or with G-CSF (filgrastim dissolved in vehicle, 30 μg/kg body weight/day) with an osmotic, subcutaneously implanted, paravertebrally located minipump (Alzet Minipump, model 2004; ALZET Osmotic Pumps, Cupertino, CA, USA) (Pitzer et al., 2008). Wild type littermates served as control group (group 3). Each mouse was housed under standard conditions in cages together with 4-6 littermates and fed *ad libitum*, in a room that also housed male mice, thus resulting in likely estrous cycle synchronization. All animal experiments were approved by the Regierungspräsidium Karlsruhe (Karlsruhe, Germany).

### Tissue isolation

All kinds of surgical instruments (forceps, scissors) were cleaned with RNase Zap spray or 100 % ethanol before use. Directions for handling RNA or tissue for RNA isolation (e.g., gloves, etc.) were followed. For anesthetizing purpose, a mixture of ketamine (120 mg/kg boday mass) and xylasine (Rompun, 16 mg/kg body mass was injected intraperitoneally. After complete absence of peripheral nociceptive reflexes the animals were perfused transcardially via the left ventricle with ice-cold HBSS. The pump was run at 10 ml/min for 3 min. Care was taken not to injure any blood vessel during the preparation. The perfused mouse was fixed onto a metal platform cooled by wet ice to avoid RNA degradation. After removal of the skin, cranial bones and vertebrae were carefully removed dorsally and spinal cords were dissected from the remaining opened cranium and vertebral canal. The thoracic position T9 was marked with a blue dye spot. Tissue was frozen on dry ice for about 5–10 min and stored at −80°C.

### Laser microdissection

Spinal cords were cryosectioned into 8 μm coronal sections, starting at the lumbar position L2. Thirty sections per mice were collected. Sections taken for microdissection were 100 μm apart. The tissue on the POL-membrane of a frame slide was overlaid with thionin staining solution so that the whole sample was completely covered. After 20 s ultraPURE™ water was poured onto the sample to wash away excess thionin. The frame slide with the tissue sample on the POL-membrane was mounted onto the microscope. Samples were collected into the cap of a 0.65 ml LMD tube filled with 35 μl of buffer RLT (Qiagen RNeasy® Micro Kit). Using the program “Leica Laser Microdissection V 5.0,” cells were marked and then automatically cut by a moving laser beam (power 40–50, speed 4, specimen/balance 11, offset 45). A total of 300 motoneurons per mouse spinal cord have been sampled by laser microdissection (Supplementary Figure 1), from a total of 35 mice (week 11 wt: *n* = 6; SOD *n* = 8); week 15 [wt *n* = 7; SOD (vehicle): *n* = 7; SOD (G-CSF treated): *n* = 7)], and subjected amplified RNA to array hybridization. Cutting was done at 40x magnification. Experimenters involved in laser microdissection were blind to the identity of the animal.

### RNA amplification

Each RNA sample was linearly amplified by two consecutive rounds of T7 polymerase-driven amplification. The precipitated total RNA of each LMD sample was reverse transcribed with a first-strand synthesis mix comprising 10 U Superscript III (Invitrogen, Karlsruhe, Germany), 1 U Superasin (Ambion, Huntingdon, UK) and 0.5 pmol of T7-T18V primer by incubating for 2 h at 50°C after second-strand synthesis with 6 U DNA polymerase I, 2 h at 22°C with 0.2 U RNaseH, and 15 min at 16°C with 4 U T4 DNA polymerase I (Invitrogen). The T7 promoter tagged cDNA template was purified by standard phenol extraction and ethanol precipitation. This cDNA was used for first round amplified antisense RNA production applying a MegaScript kit (Ambion) and purified with an RNeasy Mini Kit (Qiagen) according to the manufacturer's instructions. Second round amplification was performed accordingly but using T7-tagged random primer for first strand cDNA synthesis and T18V primer for second-strand synthesis, yielding amplified sense RNA. UV measurements at 280 and 260 nm and 2100 Bioanalyzer runs using the RNA-6000 Nano-LabChip (Agilent, Böblingen, Germany) were used to ensure the quality and quantity of the sense RNA produced. Only samples showing RNA integrity numbers (RIN) above 8 were used. The yield varied from 40 to 80 μg.

### Array analyses

The arrays were performed with the GeneChip® mouse genome 430 2.0 array from Affymetrix (Affymetrix, Santa Clara, CA, USA). Expression console software (Affymetrix, Santa Clara, CA, USA) was used to normalize arrays with the gcrma algorithm (Harr and Schlotterer, 2006) and to perform quality control. The database was filtered with intensity and standard deviation. We removed genes when one of the following conditions was not met: less than 4 arrays had intensity higher than the centile 20% of all genes or the standard deviation was lower than centile 30% of all genes.

Discriminant analysis has been performed with JMP 11.0.0 (SAS Institute, Cary, North Carolina) with genes presenting a minimum standard deviation greater than 1.2 in all individuals. Class prediction has been computed using a non-parametric k-Nearest Neighbors algorithm.

Differential gene expression among experimental groups has been assessed by ANOVA followed by a post-hoc Tukey's Honest, with genes presenting a minimum fold-change of 1.5-fold. We considered a *p*-value lower than 0.05 with a FDR lower than 10% as significant difference. Analyses were performed using BRB-ArrayTools developed by Dr. Richard Simon and BRB-ArrayTools Development Team (http://linus.nci.nih.gov).

WEB-based Gene SeT AnaLysis Toolkit was used to detect enrichment of biological functions (WebGestalt, http://bioinfo.vanderbilt.edu/webgestalt/) (Zhang et al., 2005; Wang et al., 2013).

### Real-time quantitative PCR

One μg total RNA used for the microarray was used to synthesize cDNA using superscript II reverse transcriptase (Invitrogen) and oligo-dT primers, accordingly to manufacturer's recommendation. Quantitative reverse transcription-polymerase chain reaction was performed using the Lightcycler system with SYBR-Green reagent (Roche). Cycling conditions were as follows: 10 min at 95°C; 5 s at 95°C, 10 s at the annealing temperature of 64°C, 30 s at 72°C for 50 cycles and 10 min at 95°C. Relative quantification were calculated after normalization to the reference gene cyclophilin B.

## Results

### Design of the experiment

A first group of SOD1^G93A^ and WT mice was included in the study at week 11 of age when SOD1^G93A^ mice present no signs of motor dysfunction but subtle signs of denervation detectable by electromyography (Pitzer et al., 2008). The second cohort of mice was treated with G-CSF or vehicle from week 11 to week 15. At the time of study completion, SOD1^G93A^ mice presented clear motor impairment and motoneuron degeneration is documented (Pitzer et al., 2008; Henriques et al., 2011).

During laser microdissection, there is a risk of collecting cells surrounding motoneurons and therefore analyzing non-neuronal transcripts (Supplementary Figure 1A, Perrin et al., 2005; Bandyopadhyay et al., 2013). To assess the proportion of contaminating RNA, we have studied the expression values of highly specific markers for astrocytes, oligodendrocytes, microglia, endothelial cells, and neurons (Cahoy et al., 2008). All experimental groups show high expression of the specific neuronal markers, while markers for glial and endothelial cells are present at very low levels or not detected (Supplementary Figure 1B). These data suggest that contamination with RNA from other cells than motoneurons is minimal and irrelevant for the following analyses.

**Figure 1:**
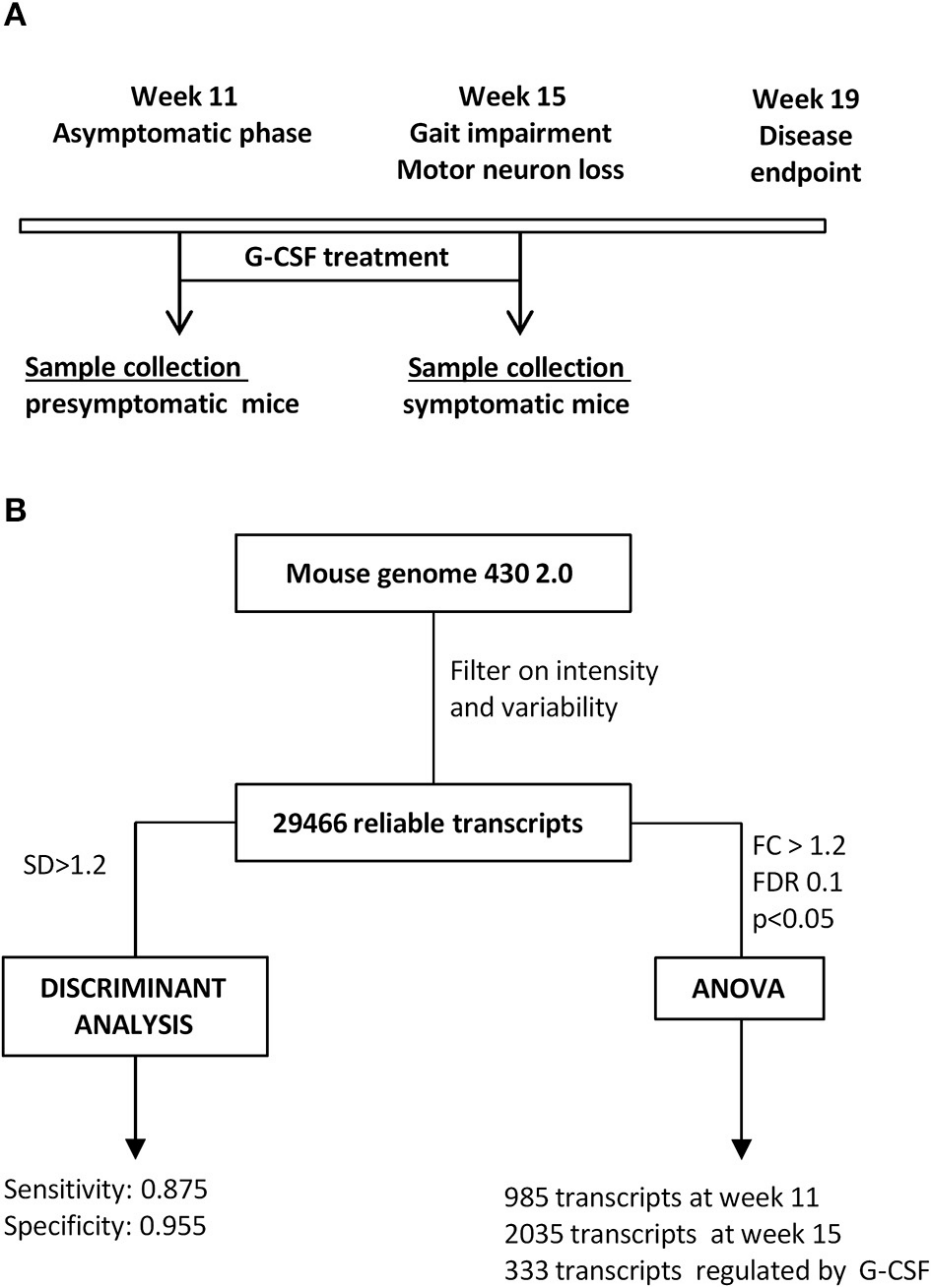
**Study design. (A)** Time course of the study. Given is the disease course in SOD1G93A mice, time of treatment and time of sacrifice for all experimental groups. **(B)** Flow chart for analytical steps for analysis.

The design of the present study should be informative on genes altered in motoneurons of SOD1^G93A^ mice from the clinically non-symptomatic to an early symptomatic stage, and give insight into genes influenced by G-CSF treatment (Figure 1A). Results were analyzed by unsupervised and supervised approaches (Figure 1B), and biological functions for relevant genes analyzed based on gene ontology.

### Gene regulation in SOD1^G93A^ motoneurons

At 11 weeks of age, when SOD1^G93A^ mice do not show motor symptoms despite early electromyographic changes we detected 985 transcripts with a significantly altered expression, of which 725 genes were upregulated and 260 dowregulated (Table 1). At 15 weeks of age, when motor symptoms are obvious and muscular denervation signs are prominent by electromyography, the number of significantly deregulated transcripts was two times higher, with 790 genes upregulated and 1245 dowregulated (Table 2). Functional annotation using gene ontology revealed that metabolic process, reponse to stimulus/stress and nucleic acid binding were the main biological and molecular functions altered in SOD-1 motoneurons (Supplementary Figure 2).

**Table 1:** **List of the main transcripts whose expression are altered in SOD1^G93A^ mice at 11 week of age**.

**Probe set ID**	**Symbol**	**Name**	**FC**	***p*-value**
1458345_s_at	Colec11	Collectin sub-family member 11	0.28	0.005
1457067_at	Vps4b	Vacuolar protein sorting 4b (yeast)	0.32	0.016
1444869_at	–	–	0.34	0.001
1427351_s_at	Ighm	Immunoglobulin heavy constant mu	0.34	0.000
1419332_at	Egfl6	EGF-like-domain, multiple 6	0.38	0.000
1423608_at	Itm2a	Integral membrane protein 2A	0.38	0.000
1438763_at	Dnahc2	Dynein, axonemal, heavy chain 2	0.39	0.000
1435815_at	Ldoc1	Leucine zipper, down-regulated in cancer 1	0.40	0.000
1423390_at	Siah1a	Seven in absentia 1A	0.41	0.000
1418916_a_at	Spp2	Secreted phosphoprotein 2	0.43	0.000
1441114_at	9330156P08Rik	RIKEN cDNA 9330156P08 gene	0.44	0.004
1420386_at	Seh1l	SEH1-like	0.45	0.005
1419435_at	Aox1	Aldehyde oxidase 1	0.46	0.001
1445767_at	Ptprd	Protein tyrosine phosphatase, receptor type, D	0.46	0.001
1452276_at	Smarcad1	SWI/SNF-related, matrix-associated actin-dependent regulator of chromatin, subfamy a, containing DEAD/H box1	0.46	0.013
1437796_at	–	–	0.46	0.000
1427196_at	Wnk4	WNK lysine deficient protein kinase 4	0.46	0.016
1451440_at	Chodl	Chondrolectin	0.46	0.000
1439011_at	Gm20245	Predicted gene, 20245	0.46	0.044
1438143_s_at	Atxn2	Ataxin 2	0.47	0.000
1449662_at	Reep1	Receptor accessory protein 1	4.0	0.0000
1434442_at	Stbd1	Starch binding domain 1	4.0	0.0001
1426851_a_at	Nov	Nephroblastoma overexpressed gene	4.1	0.0003
1426808_at	Lgals3	Lectin, galactose binding, soluble 3	4.2	0.0000
1440142_s_at	Gfap	Glial fibrillary acidic protein	4.4	0.0000
1419665_a_at	Nupr1	Nuclear protein 1	4.5	0.0000
1423427_at	Adcyap1	Adenylate cyclase activating polypeptide 1	4.9	0.0002
1449519_at	Gadd45a	Growth arrest and DNA-damage-inducible 45 alpha	5.1	0.0000
1417022_at	Slc7a3	Solute carrier family 7, member3	5.1	0.0000
1451285_at	Fus	Fusion, derived from t(12;16) malignant liposarcoma (human)	5.6	0.0060
1417868_a_at	Ctsz	cathepsin Z	5.7	0.0000
1422916_at	Fgf21	Fibroblast growth factor 21	6.1	0.0000
1437621_x_at	Phgdh	3-phosphoglycerate dehydrogenase	8.3	0.0000
1437232_at	Bpifc	BPI fold containing family C	8.4	0.0000
1454714_x_at	Phgdh	3-phosphoglycerate dehydrogenase	8.7	0.0000
1434129_s_at	Lhfpl2	Lipoma HMGIC fusion partner-like 2	10.6	0.0000
1449153_at	Mmp12	Matrix metallopeptidase 12	12.3	0.0002
1449133_at	Sprr1a	small proline-rich protein 1A	13.7	0.0000
1449363_at	Atf3	activating transcription factor 3	14.4	0.0000

**Table 2:** **List of the main transcripts whose expression are altered in SOD1^G93A^ mice at 15 week of age**.

**Probe set ID**	**Symbol**	**Name**	**FC**	***p*-value**
1457587_at	Kcnq5	Potassium voltage-gated channel, subfamily Q, member5	0.08	0
1423608_at	Itm2a	Integral membrane protein 2A	0.1	0
1427329_a_at	Ighm	Immunoglobulin heavy constant mu	0.12	0
1427351_s_at	Ighm	Immunoglobulin heavy constant mu	0.13	0
1451440_at	Chodl	Chondrolectin	0.13	0
1451047_at	Itm2a	Integral membrane protein 2A	0.14	0
1429759_at	Rps6ka6	Ribosomal protein S6 kinase polypeptide 6	0.15	0
1445268_at	–	–	0.15	0
1455291_s_at	Znrf2	Zinc and ring finger 2	0.17	0.001
1455238_at	Mum1l1	Melanoma associated antigen (mutated) 1-like 1	0.17	0
1449155_at	Polr3g	Polymerase (RNA) III (DNA directed) polypeptide G	0.17	0
1433898_at	–	–	0.17	0
1441801_at	Kctd4	Potassium channel tetramerisation domain containing 4	0.18	0
1452366_at	Csgalnact1	Chondroitin sulfate n-acetylgalactosaminyltransferase 1	0.18	0
1418469_at	Nrip1	Nuclear receptor interacting protein 1	0.18	0.005
1434102_at	Nfib	Nuclear factor i/b	0.18	0
1433827_at	Atp8a1	ATPase, aminophospholipid transporter (APLT), class I, type 8A, member 1	0.19	0
1438214_at	Trps1	Trichorhinophalangeal syndrome I (human)	0.19	0.003
1422880_at	Sypl	Synaptophysin-like protein	0.19	0
1460227_at	Timp1	Tissue inhibitor of metalloproteinase 1	4.8	0.0001
1434376_at	Cd44	CD44 antigen	5.2	0.0013
1434129_s_at	Lhfpl2	Lipoma HMGIC fusion partner-like 2	5.6	0
1423427_at	Adcyap1	Adenylate cyclase activating polypeptide 1	5.6	0.0002
1426852_x_at	Nov	Nephroblastoma overexpressed gene	5.9	0
1448471_a_at	Ctla2a	Cytotoxic T lymphocyte-associated protein 2 alpha	5.9	0.0065
1429051_s_at	Sox11	SRY-box containing gene 11	6	0.0003
1454714_x_at	Phgdh	3-phosphoglycerate dehydrogenase	6.4	0
1437726_x_at	C1qb	Complement component 1, q subcomponent, beta polypeptide	6.6	0
1436996_x_at	Lyz1	Lysozyme 1	6.7	0.0054
1450792_at	Tyrobp	TYRO protein tyrosine kinase binding protein	7.1	0.0002
1437621_x_at	Phgdh	3-phosphoglycerate dehydrogenase	7.8	0
1423760_at	Cd44	CD44 antigen	7.9	0.0003
1434046_at	AA467197	Expressed sequence AA467197	8.3	0.0001
1426808_at	Lgals3	Lectin, galactose binding, soluble 3	8.9	0
1423537_at	Gap43	Growth associated protein 43	8.9	0
1449363_at	Atf3	Activating transcription factor 3	10	0
1440142_s_at	Gfap	Glial fibrillary acidic protein	13	0
1449133_at	Sprr1a	Small proline-rich protein 1A	20.6	0

At the presymptomatic disease stage (week 11), genes which were most downregulated were the collectin sub-family member 11 (colec11) and vacuolar protein sorting 4b (vpsb4) (Table 1). Expression of colec11 has been reported in neurons and is involved in innate immunity (Motomura et al., 2008; Ohtani et al., 2012). vpsb4 is known to interact with the pro-apoptotic pathway (Cui et al., 2014) and the autophagosome in neurodegenerative conditions (Han et al., 2012). The most strongly upregulated genes were related to response to stress and injury, such as activating transcription factor 3 (atf3) and small proline-rich repeat protein 1A (sprr1A). Upregulation of atf3 has already been noted in motoneurons after axotomy (Tsujino et al., 2000) and in response to deficiency in neurotrophic factors (Hyatt Sachs et al., 2007). Sprr1A was the second most stimulated gene and, similar to atf3, its expression is enhanced in motoneurons upon axotomy in order to promote axonal growth (Bonilla et al., 2002; Li and Strittmatter, 2003; Starkey et al., 2009). Interestingly, the fus (fused in sarcoma) gene was strongly upregulated in the presymtomatic disease stage. Fus protein is a RNA-binding protein and has important functions in RNA metabolism (e.g. splicing, formation of stress granule, transport and local translation). Mutations in fus are found in familial forms of ALS, and inclusions of the protein are present in patients with frontotemporal dementia, a syndrome that is connected to ALS (Ling et al., 2013).

At the symptomatic disease stage, the most overexpressed genes were mainly associated with motoneuron stress response and support of axonal regeneration, similar to the presymptomatic disease stage (Table 2). Beside sprr1A and atf3, the growth associated protein 43 (gap43) shows increased expression (9-fold) which is involved in axonal regeneration after motoneuron injury (Chen et al., 2010; Grasselli et al., 2011). Interestingly, the transcription factor responsible for sprr1A expression in motoneurons, sox11, is strongly upregulated at this disease stage. Potassium voltage-gated channel, subfamily Q, member 5 (kcnq5) was the most downregulated gene (downregulated 14-fold) at the symptomatic disease stage. Its function in motoneurons is not described, but should be related to the control of resting and release properties of synapses (Huang and Trussell, 2011). The expression of chondrolectin (chodl) was also strongly repressed in motoneurons of SOD1^G93A^ mice. Interestingly, this protein promotes motor axon growth and growth cone interactions, and its downregulation is associated with motoneuron degeneration in an animal model of spinal muscular atrophy (Zhong et al., 2012; Sleigh et al., 2014). As shown in Supplementary Table 1, 83 transcripts were commonly deregulated at both asymptomatic and symptomatic disease stages. We noted deregulation at the two disease stages for several growth factors (FGF21, VGF, TGFa), and genes related to DNA/RNA metabolism (wars, eef1a1, eif4e).

### G-CSF partially restores the motoneuron transcriptional signature in SOD1^G93A^ mice

In order to better understand the effects of G-CSF in the SOD1^G93A^ mice, we have monitored transcriptional changes in motoneurons at a symptomatic disease stage upon a 4 weeks treatment (from week 11 to week 15). We performed a discriminant analysis with transcripts showing a standard deviation greater than 1.2 across all samples (734 transcripts).

**Figure 2:**
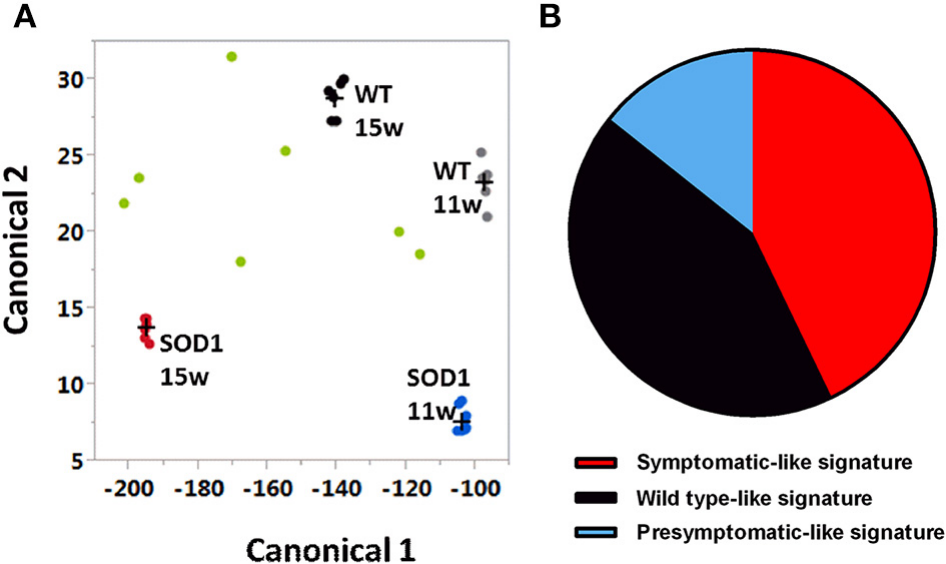
**Canonical plot shows dispersion of treated animals off the non-treated groups**. Multivariate analyses have been conducted with genes having a standard deviation greater than 1.2 (734 transcripts). **(A)** Canonical plot shows that symptomatic SOD-1 mice (red dots) are distant from other groups, including asymptomatic SOD1 mice (blue dots) and when considered as unknown, SOD-1 mice treated with G-CSF (green dots) show a strong dispersion and tend to be close to wild types at 11 weeks (gray dots) or 15 weeks of age (black dots). **(B)** After G-CSF treatment, discriminant analysis classified only three samples of SOD1 motorneurons as symptomatic motor neurons. One sample was considered has presymptomatic and three as wild type motor neurons.

As shown in a canonical plot, control groups are close to each other and SOD1^G93A^ mice are distant from non-transgenic animals (Figure 2A). The SOD1 mice treated with G-CSF (green dots in Figure 2) tended to have a transcriptomic signature different from the sympatomatic SOD1 mice (red dots in Figure 2), that was closer to non-symptomatic mice. This observation was further confirmed by class prediction using a non-parametric k-Nearest Neighbors algorithm, used to predict the class of unknown samples. The algorithm was built with the signatures of all samples with the exclusion of mice treated with G-CSF. There were 4 defined classes: wild type 11 weeks, SOD-1^G93A^ 11 weeks (presymptomatic), wild type 15 weeks and SOD-1^G93A^ mice 15 weeks (symptomatic). SOD-1^G93A^ mice treated with G-CSF were considered as unknown and the algorithm sorted them into the defined classes based on their transcriptome signature. The mean percent of correct classification reached 96% of efficacy for the defined classes, with a sensitivity above 0.875 and a specificity of 0.955.

The group treated with G-CSF was composed by seven SOD-1^G93A^ mice. Three out of seven were sorted as symptomatic SOD-1^G93A^, one as asymptomatic SOD-1^G93A^ 11 weeks, and three as wild type 15 weeks (Figure 2B). These data suggest that G-CSF can modulate the gene expression in motoneurons of 15 weeks old SOD-1^G93A^ mice and can prevent disease-related changes to approach the transcriptomic signature of healthy mice or asymptomatic SOD1^G93A^ animals.

### Genes regulated by G-CSF in motoneurons of SOD1^G93A^ mice

**Figure 3:**
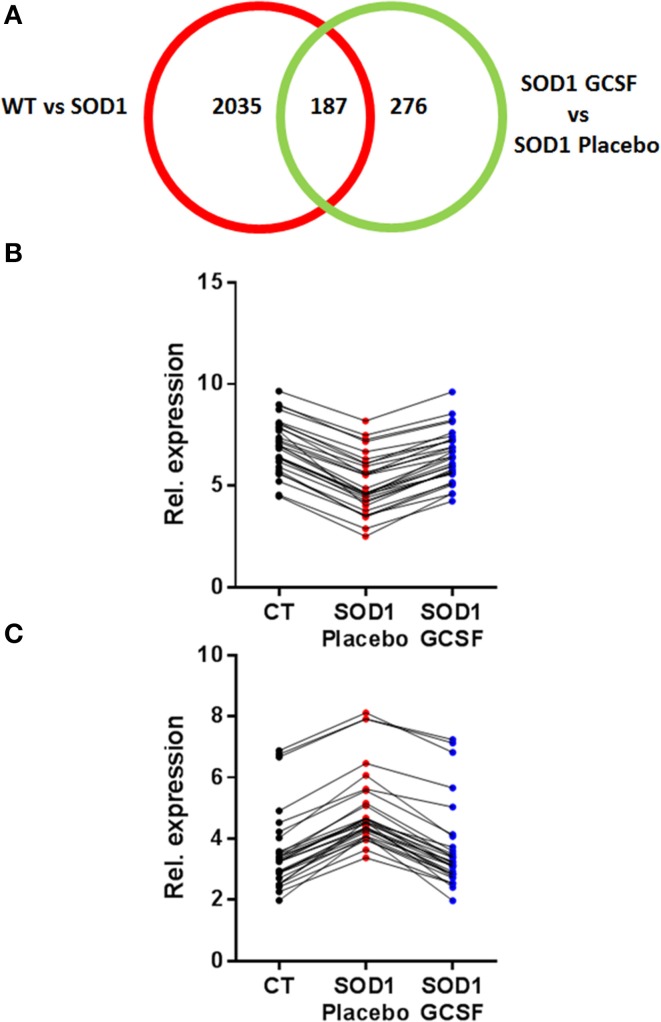
**G-CSF treatment modulates transgene-related gene expression changes. (A)** Chart showing the number of differentially regulated genes after G-CSF treatment in the motoneurons of SOD1 mice. **(B–C)** Given is the relative expression of the 30 most significantly modified transcripts re-adjusted by G-CSF treatment and previously found downregulated **(B)** or upregulated **(C)** in symptomatic SOD1 mice.

Next, we focused on the identity of differentially expressed genes in motoneurons after G-CSF treatment. G-CSF had an effect on the regulation of 463 transcripts in total, and was able to adjust the expression of 187 transcripts that were either up- or downregulated in SOD1^G93A^ mice compared to controls (Tables 3, 4, Figure 3).

**Table 3:** **List of the main transcripts whose expression is downregulated by G-CSF treatment in SOD1^G93A^ mice at 15 week of age**.

**Probe set ID**	**Gene symbol**	***p*-value**	**WT**	**SOD1**	**SOD1 G-CSF**	**FC WTvsSOD1**	**FC WT vs. SOD1-GCSF**
1421979_at	Phex	0.000	1.994	4.091	1.983	4.279	0.992
1421316_at	Lce1g	0.001	2.505	4.563	2.415	4.167	0.940
1419127_at	Npy	0.000	4.025	6.074	4.075	4.138	1.035
1456084_x_at	Fmod	0.000	2.536	4.400	2.752	3.640	1.161
1437720_at	Eif2d	0.000	4.914	6.458	5.661	2.916	1.679
1438856_x_at	Serpinb5	0.018	2.880	4.356	2.842	2.781	0.974
1441836_x_at	Gtsf1	0.003	2.524	3.958	2.718	2.703	1.144
1444780_at	Nav2	0.001	3.251	4.684	3.440	2.700	1.140
1418778_at	Ccdc109b	0.001	3.279	4.642	3.071	2.571	0.865
1437775_at	Dlst	0.003	4.229	5.570	4.142	2.532	0.941
1448048_at	Nmrk1	0.006	2.889	4.219	2.927	2.515	1.027
1458056_at	Srek1	0.003	2.954	4.280	3.260	2.507	1.236
1454557_at	6720454L07Rik	0.000	3.338	4.651	3.503	2.485	1.122
1417491_at	Ctsb	0.000	6.668	7.909	7.128	2.364	1.376
1436242_a_at	Cklf	0.019	6.877	8.107	6.819	2.346	0.961
1458335_x_at	Urm1	0.000	6.757	7.911	7.240	2.225	1.398
1428514_at	Cpne3	0.000	2.936	4.085	3.124	2.218	1.139
1456816_at	Zmiz1	0.001	3.413	4.530	3.732	2.170	1.248
1433733_a_at	Cry1	0.001	2.270	3.378	2.549	2.155	1.213
1418467_at	Smarcd3	0.000	4.533	5.617	5.038	2.121	1.419
1429688_at	Arntl2	0.014	2.893	3.971	2.846	2.111	0.968
1460578_at	Fgd5	0.000	3.593	4.669	3.192	2.108	0.757
1457147_at	Etl4	0.001	3.500	4.570	3.118	2.099	0.767
1437822_at	Yme1l1	0.004	3.426	4.485	3.395	2.084	0.979
1458669_at	Agpat5	0.003	3.253	4.304	3.132	2.073	0.919
1443883_at	Sys1	0.000	4.305	5.316	4.112	2.015	0.875
1433882_at	Cnot10	0.000	5.577	6.566	5.363	1.985	0.862
1437718_x_at	Fmod	0.000	2.890	3.862	2.837	1.961	0.964
1440398_at		0.001	2.641	3.608	2.866	1.954	1.169
1445861_at	Usp25	0.014	3.428	4.393	3.399	1.952	0.980
1443242_at	D5Ertd121e	0.005	2.186	3.150	2.205	1.950	1.013

**Table 4:** **List of the main transcripts whose expression is upregulated by G-CSF treatment in SOD1^G93A^ mice at 15 week of age**.

**Probe set ID**	**Gene symbol**	***p*-value**	**WT**	**SOD1**	**SOD1 G-CSF**	**FC WTvsSOD1**	**FC WT vs. SOD1-GCSF**
1457587_at	Kcnq5	0.000	7.713	4.026	5.814	0.078	0.268
1455102_at	Larp4	0.000	6.999	4.642	6.371	0.195	0.647
1455381_at	Marf1	0.002	7.906	5.585	7.319	0.200	0.666
1449861_at	Nek4	0.001	5.754	3.478	5.055	0.207	0.616
1460440_at	Lphn3	0.000	6.848	4.670	6.104	0.221	0.597
1419555_at	Elf5	0.000	5.646	3.526	5.185	0.230	0.727
1455960_at	Megf9	0.000	6.959	4.882	6.446	0.237	0.701
1429475_at	Ubash3b	0.001	6.351	4.279	5.680	0.238	0.628
1420388_at	Prss12	0.000	7.504	5.467	6.385	0.244	0.461
1449348_at	Mpp6	0.000	8.104	6.114	7.248	0.252	0.552
1438435_at	Acer3	0.001	4.492	2.529	4.626	0.257	1.098
1429107_at	Ubr3	0.001	6.415	4.532	5.996	0.271	0.748
1451217_a_at	Immp1l	0.000	6.378	4.530	6.677	0.278	1.230
1419357_at	Isy1	0.000	5.599	3.778	5.114	0.283	0.715
1423597_at	Atp8a1	0.000	8.998	7.187	8.151	0.285	0.556
1418973_at	Blzf1	0.000	6.188	4.426	5.637	0.295	0.683
1454937_at	B630005N14Rik	0.000	6.420	4.661	5.956	0.295	0.725
1435224_at	Crebbp	0.015	7.833	6.076	7.621	0.296	0.863
1455728_at	Pten	0.001	5.930	4.204	5.737	0.302	0.875
1416468_at	Aldh1a1	0.000	8.034	6.314	7.206	0.304	0.563
1425493_at	Bmpr1a	0.000	4.559	2.907	4.253	0.318	0.809
1417564_at	Med7	0.000	7.151	5.551	6.891	0.330	0.835
1423298_at	Add3	0.000	7.243	5.681	6.749	0.339	0.710
1421904_at	Tgs1	0.001	8.757	7.283	8.236	0.360	0.697
1416655_at	C1galt1c1	0.001	9.662	8.190	9.617	0.360	0.969
1460409_at	Cpt1a	0.000	8.140	6.686	7.440	0.365	0.616
1451308_at	Elovl4	0.000	6.986	5.539	6.436	0.367	0.683
1423490_at	Fbxo3	0.000	8.932	7.495	8.538	0.369	0.761
1455807_at	Tspyl5	0.001	7.369	5.965	6.909	0.378	0.727

Two of the most downregulated transcripts in SOD1^G93A^ mice, kcnq5 and larp4, had their expression enhanced by a factor 3 upon G-CSF treatment as compared to placebo group. Larp4 has no known specific functions in neurons but is known to regulates mRNA stability (Yang et al., 2011). G-CSF prevented the upregulation of NPY, a well-known neuropeptide involved in energetic metabolism and food intake but also expressed by neurons in response to stress such as spinal cord inflammation (Taylor et al., 2014). Upregulation of cklf, a cytokine with lymphocyte chemoattractive properties, was also inhibited by treatment (Han et al., 2001). Interestingly, Cpt1a and elovl4, two transcripts directly associated with lipid metabolism, were downregulated in SOD1^G93A^ motoneurons, and upregulated by G-CSF. Cpt1a controls the entrance of fatty acids into mitochondria and is therefore the rate limiting enzyme for fatty acid oxidation, and Elovl4 elongates long fatty acids without distinction on their saturation levels, suggesting that G-CSF could enhance the processing of fatty acids in spinal cord. Additionally, G-CSF modulated the expression of 251 previously unaltered transcripts. The effect of G-CSF was prominent on gene ontology biological functions deregulated in SOD1^G93A^ mice, including metabolic process, and response to stimulus (Supplementary Figure 2).

In order to assess the reliability of the analysis, we selected several genes for validation by quantitative real time PCR (qPCR). Those genes were selected because of their altered expression in SOD1^G93A^ mice at 15 weeks of age and by a significant effect of G-CSF on their regulation. An additional criterion was their potential role in the neurodegenerative process of ALS based on the literature. We chose to study the regulation of CNOT10, CTSB, Prss12 and BMPR1a. CNOT10 is a member of the CCR4-NOT complex that acts as a chaperon platform (Collart and Panasenko, 2012). The CCR4-NOT complex controls and promotes neuromuscular junction formation in drosophila (Pradhan et al., 2012). Prss12, also known as motopsin, is a protease specific to neurons and its expression is downregulated upon stress, such as axotomy, in motoneurons (Numajiri et al., 2009). Its regulation is therefore seen as a marker of denervation. We chose ctsb, as meta-analysis of transcriptomic studies in ALS have shown its expression systematically altered, both in human patients and SOD1^G93A^ mice (Saris et al., 2013). Ctsb codes for cathepsin B, a protein associated with neuroinflammation, motoneuron degeneration, and tissue destruction (Kikuchi et al., 2003). BMPR1a is a receptor serine/threonine kinase and regulates apoptosis and cell differentiation (Mishina et al., 2004). In the central nervous system, BMP signaling is required during formation of large-transmitting synapses (Xiao et al., 2013), build-up of neuromuscular junctions (Higashi-Kovtun et al., 2010) and is stimulated upon spinal cord and sciatic nerve injury (Tsujii et al., 2009; Sahni et al., 2010).

**Figure 4:**
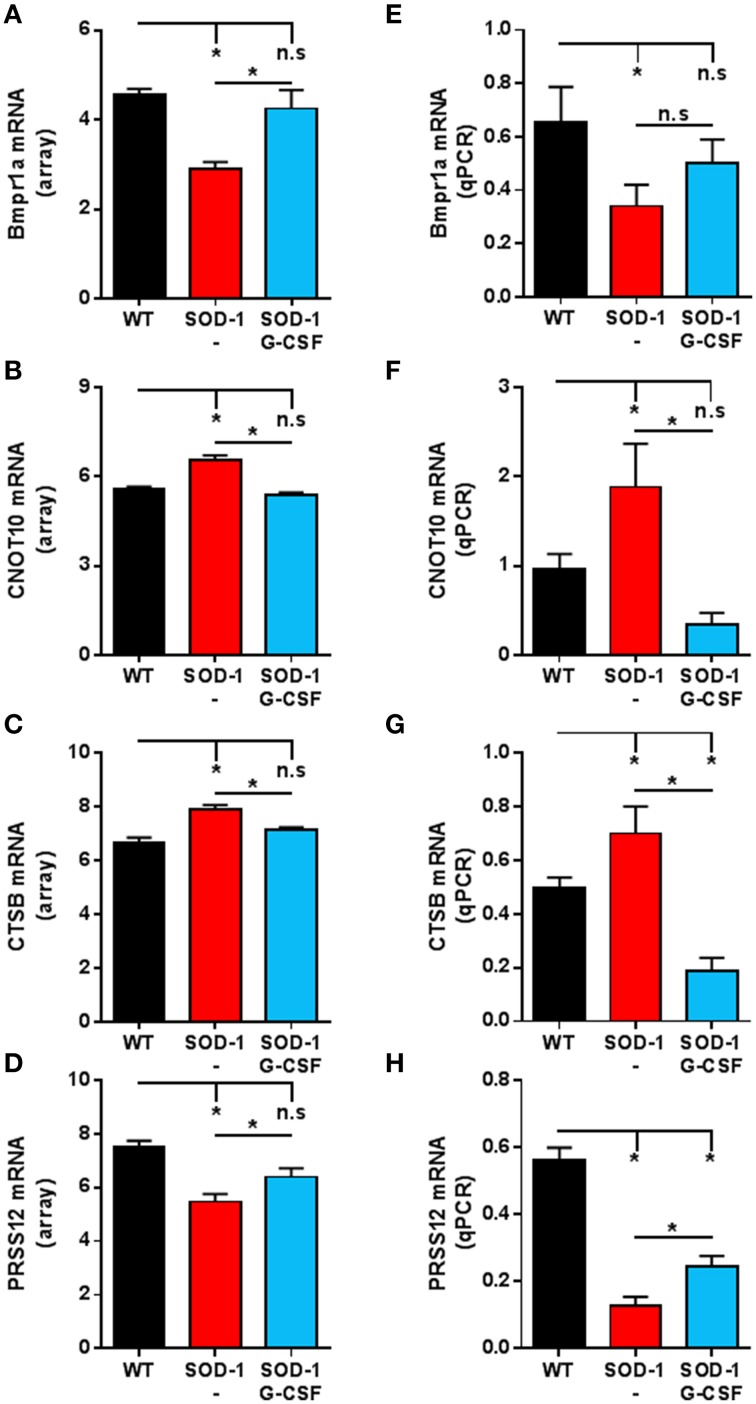
**Validation of the expression of selected transcripts by qPCR. (A–D)** Expression levels from microarray analyses of Bmpr1a **(A)**, CNOT10 **(B)**, CTSB **(C)** and PRSS12 **(D)** at the symptomatic disease stage. **(E–H)** Expression levels derived from quantitative PCR of Bmpr1a **(E)**, CNOT10 **(F)**, CTSB **(G)** and PRSS12 **(H)** at the symptomatic disease stage. Data are presented as mean ± standard variation of the mean. ^*^p < 0.05 (ANOVA, followed by multicomparison *t*-test).

Relative expressions for these transcripts were highly similar to the relative abundance derived from microarray analyses (Figure 4). In particular, upregulation of CNOT10 present in SOD1^G93A^ mice is completely reversed upon G-CSF treatment. The regulation of bmpr1a showed a trend to the wild type value but this was not significant in the qPCR analysis.

Taken together, these data demonstrate that G-CSF promotes transcriptional changes in motoneurons in symptomatic SOD1^G93A^ mice and can correct the alterations of many disease related gene expression changes.

## Discussion

ALS is a fatal condition characterized by the constant death of motoneurons. Although the triggering factors for ALS remain unknown, the step that induces symptoms is the disruption of the motor units, with the dismantlement of neuromuscular junctions and motoneuron stress and death. The study of global changes in the transcriptome of specific cells in the motor axis is a promising approach to address the complexity of ALS (Henriques and Gonzalez De Aguilar, 2011).

### Transcriptomic changes of motoneurons from SOD1^G93A^ mice

Here, we studied the transcriptome of motoneurons of SOD1^G93A^ mice, at two disease stages and after a pharmacological treatment with G-CSF, a trophic factor with neuroprotective properties (Pitzer et al., 2008; Henriques et al., 2010a,b, 2011). Our aim was to characterize the transcriptomic reponse of motor neurons to a neuroprotective treatment based on G-CSF.

In 2005, a first study was published describing the transcriptome of laser microdissected motoneurons of SOD1^G93A^ mice at three different disease stages (Perrin et al., 2005). The conclusion of the authors was that few changes occurred at presymptomatic (28 deregulated transcripts) and early symptomatic (148 deregulated transcripts) stages, and that symptomatic stages were characterized by few expression changes in cell-death-associated genes. They found deregulation of related to energetic metabolism, cell growth and/or maintenance (Perrin et al., 2005). Many of these genes also showed an altered expression in our study. In 2007, another group published data on the transcriptome of dissected motoneurons from SOD1^G93A^ mice, and reported dysregulations on genes related to transcription and nuclear proteins, protein synthesis, metabolism and cell cycle/growth (Ferraiuolo et al., 2007). Both studies identified lower deregulation events than our study. This difference can most likely be explained by higher statistical power in our study as both studies used fewer animals (*n* = 3/group) than our study (at least n = 6 per group). Higher degree of contaminating RNA from motoneuron-surrounding cells could also account for the differences between studies.

Based on our results, the transcriptome of isolated motoneurons of SOD1^G93A^ mice is characterized by the deregulation of genes, starting at presymptomatic disease stages, related to response to stress, to regulation of transcription and to metabolic processes.

Response to stress is stimulated early on, at presymptomatic disease stage. Trophic signaling seems also to be stimulated, with the upregulation of the trophic factors such as fgf21 and vgf at presymptomatic and symptomatic disease stages. FGF21 belongs to the fibroblast growth factor family, known to counteract motoneuron death after axotomy-induced apoptosis (Cuevas et al., 1995), but also shown to regulate activation of astrocytes in animal models of ALS (Cassina et al., 2005). Additionally, motoneurons of SOD1^G93A^ show an upregulation of transcription factors related to stress response, that are also closely connected to trophic factors, such as ATF3 (Averill et al., 2004).

During laser-microdissection of motoneurons, small amount of astrocyte RNA may have been captured. Although this contamination is minimal in our study, genes that are expressed at low abundance in motoneurons but at high levels in glial cells could be detected as differentially regulated, provided that the difference between two groups is very strong. An indication for that could be the detection of GFAP among the deregulatated transcripts in our study (upregulated in SOD1 mice). Presence of transcripts of glial origin in studies working on laser-captured motoneurons has been described previously (Perrin et al., 2005; Ferraiuolo et al., 2007). Therefore, it is possible that the alterations in the transcriptome linked to energetic/lipid metabolism arise from astrocytes surrounding motoneurons. G-CSF was indeed interestingly able to stimulate the expression of cpt1a in SOD1^G93A^ mice, which is surprising since fatty acids are not the main energetic source for neurons. It is therefore possible that upregulation of cpt1a takes place in astrocytes and not in motoneurons, presumably to sustain higher energetic needs for neurons. Indeed, during energetic stress, astrocytes are able to convert lipid to ketone bodies that will be used as energetic fuel by surrounding neurons (Guzman and Blazquez, 2004). Interestingly, recent studies have shown increased ketone bodies in the cerebrospinal fluid of patients and fatty acid and ketone body supplementations have neuroprotective effects on motoneurons in animal models of ALS (Blasco et al., 2010; Schmitt et al., 2014). Energetic metabolism represents one promising field of research in ALS. Patients present with hypermetabolism, higher energetic needs and a higher level of circulating lipids is associated with better survival (Schmitt et al., 2014). Two recent clinical reports strongly suggest benefit after high lipid/caloric diets in ALS patients with a strong effect on survival (Dorst et al., 2013; Wills et al., 2014).

We found deregulation of several genes associated to RNA metabolism, such as Wars, Eef1a1 and Eif4e. Growing evidence suggests a role for defect RNA processing in motoneuron death. ALS patients present pathological inclusion of TDP-43 and FUS, two proteins that closely interact with RNA metabolism (Ling et al., 2013). Mutations in TARDBP (coding for TDP-43) and FUS are described in ALS patients with a family history but also in patients with fronto-temporal dementia (FTD) with a high risk (around 30%) to develop ALS. Expanded repeats in the gene C9orf72 are largely present in sporadic and familial ALS and FTD cases. Interestingly, whose expanded repeats are believed to induce RNA toxicity through a novel negative gain-of-function. Our results therefore provide interesting new clues when related to most recent pathophysiological hypotheses in ALS research.

### G-CSF action on motoneurons

Our transcriptomic study describes for the first time the transcriptomic changes in motoneurons after a neuroprotective/ -regenerative treatment. We chose to perform our study with SOD1^G93A^ mice at 15 week of age with an administration of G-CSF for 4 weeks. At this age, motor symptom are clearly established in untreated animals (Pitzer et al., 2008; Henriques et al., 2011) and G-CSF has a significant beneficial effect on motor functions and preserves motoneuron counts (Pitzer et al., 2008). In our study, the altered transcriptome of SOD1^G93A^ alpha motoneurons has been modified by G-CSF and the expressions of 187 genes were partially restored. Genes involved in the maintenance of the motor-units, such as Prss12 and CNOT10, were modulated upon treatment, but effect of G-CSF was also observed on other different molecular functions such as neuroinflammation, RNA processing, and energetic metabolism. Completely unsupervised analyses identified more than half of treated mice as asymptomatic based on their transcriptomic profile, confirming the positive effect of the treatment on disease progression.

Modification of the transcriptom was most likely due to a direct effect of G-CSF on the motor neurons. Motoneurons express the receptor of G-CSF in basal condition and can upregulate it after an acute stress in neonatal age (Pitzer et al., 2008; Henriques et al., 2010a). We have also demonstrated that G-CSF can rescue motoneurons in a pure apoptotic condition, induced by sciatic nerve axotomy (Henriques et al., 2010a) and promotes reinnervation after sciatic nerve crush (Henriques et al., 2011). However, we cannot formally conclude whether all or most of the observed changes are due to direct actions of G-CSF on motoneurons or reflect improved health status of the alpha motoneurons by indirect treatment effects. Indeed, G-CSF was first described as a hematopoietic growth factor involved in the proliferation and differentiation of neutrophils. Despite an abundant literature on the involvement of neuroinflammation in ALS (Philips and Robberecht, 2011), there is no evidence that neutrophils have a role in the initiation or progression of ALS, neither in animal model nor in ALS patients. Additionally, a recent report suggests that G-CSF might be involved in response to muscle damage, which is also a characteristic of ALS(Kruger et al., 2014).

## Conclusion

G-CSF was able to re-adjust the expression of genes important for motoneuron survival and function but also genes from other physiological pathways. The global changes occurring after treatment strengthens the notion of multimodal activity of G-CSF for preservation of motor units in ALS. Our results complement the existing data on characterization of G-CSF's action in mouse ALS models (Pitzer et al., 2008; Henriques et al., 2010a, 2011; Naumenko et al., 2011; Pollari et al., 2011) on the gene expression level. This increases confidence in G-CSF as a promising drug candidate for ALS.

## Author contributions

Armin Schneider designed the study. Stefan Kastner, Eva Chatzikonstantinou, Friederike Kirsch, Carola Krüger, Christian Plaas performed animal experiments, histological work, laser microdissection, RNA preparation and amplification. Norbert Gretz performed array hybridizations. Alexandre Henriques performed data analysis. Armin Schneider, Alexandre Henriques, Stefan Kastner, Claudia Pitzer, Carola Krüger, Robert Spoelgen, Jose-Luis Gonzalez De Aguilar and Armin Schneider were involved in data evaluation and discussions. Alexandre Henriques and Armin Schneider wrote the manuscript. All authors read and approved the final manuscript.

### Conflict of interest statement

Some authors are employees of Sygnis Bioscience. Armin Schneider is inventor on patent applications claiming the use of G-CSF for the treatment of neurodegenerative conditions. The authors declare that the research was conducted in the absence of any commercial or financial relationships that could be construed as a potential conflict of interest.

## Supplementary material

Supplementary Figure 1 **Laser microdissection and neuronal specificity. (A)** Representative pictures of spinal cord section before and after laser capture. Sections are stained with thionin. **(B)** Given is the relative expression of genes that are accepted as specific markers of astrocytes (*n* = 6 markers), neurons (*n* = 7 markers), oligodendrocytes (*n* = 7), microglia (*n* = 4) and endothelial cells (*n* = 4).

Supplementary Figure 2 **Biological and molecular functions of genes altered in SOD1^G93A^ mice**. Given are the main biological **(A)** and molecular **(B)** functions of genes altered in SOD1^G93A^ motoneurons at presymptomatic and symptomatic disease stages, and re-adjusted after G-CSF treatment.

Availability of Supporting Data Microarray data files have been uploaded to the Gene Expression Omnibus (GEO) database repository, under the accession number GSE60856 (www.ncbi.nlm.nih.gov/geo/).
