# Supplementary figures and images for: Gene expression changes in spinal motoneurons of the SOD1G93A transgenic model for ALS after treatment with G-CSF
Source: Front Cell Neurosci. 2015 Jan 20;8:464. doi: 10.3389/fncel.2014.00464 (PMC4299451; doi:10.3389/fncel.2014.00464)

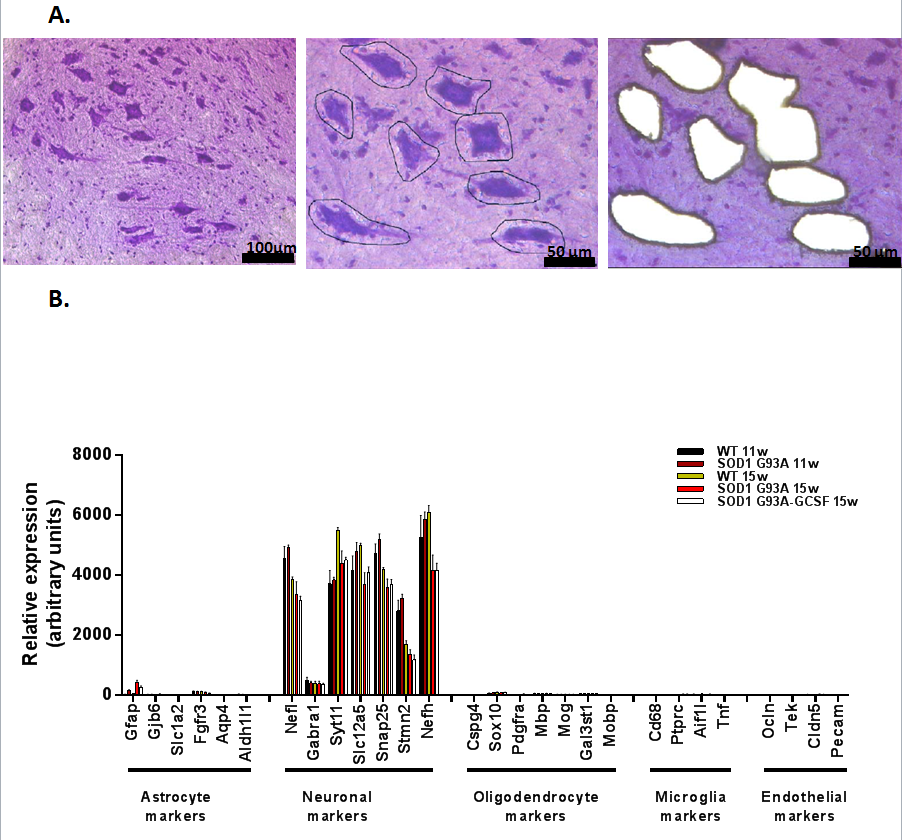

Supplement: Supplementary Figure 1 — Laser microdissection and neuronal specificity. (A) Representative pictures of spinal cord section before and after laser capture. Sections are stained with thionin. (B) Given is the relative expression of genes that are accepted as specific markers of astrocytes (n = 6 markers), neurons (n = 7 markers), oligodendrocytes (n = 7), microglia (n = 4) and endothelial cells (n = 4). [file Image1.TIF]

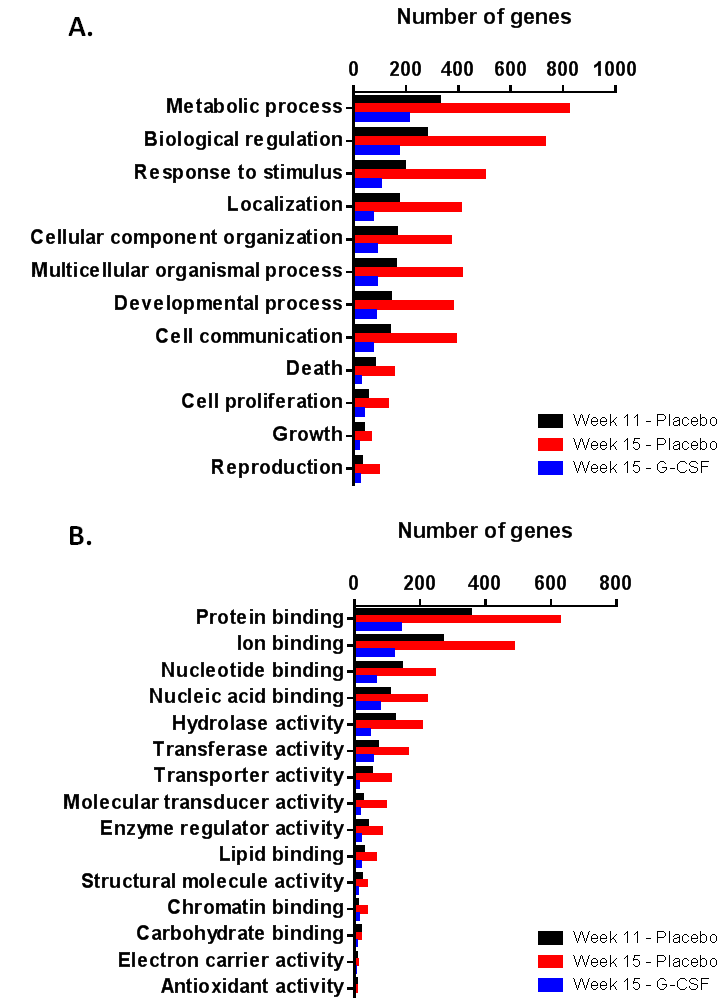

Supplement: Supplementary Figure 2 — Biological and molecular functions of genes altered in SOD1G93A mice. Given are the main biological (A) and molecular (B) functions of genes altered in SOD1G93A motoneurons at presymptomatic and symptomatic disease stages, and re-adjusted after G-CSF treatment. [file Image2.TIF]
