# Supplementary material for: Gene expression changes in spinal motoneurons of the SOD1G93A transgenic model for ALS after treatment with G-CSF
Source: Front Cell Neurosci. 2015 Jan 20;8:464. doi: 10.3389/fncel.2014.00464 (PMC4299451; doi:10.3389/fncel.2014.00464)
Supplement: Availability of Supporting Data — Microarray data files have been uploaded to the Gene Expression Omnibus (GEO) database repository, under the accession number GSE60856 (www.ncbi.nlm.nih.gov/geo/). [file DataSheet1.DOCX]

**Supplementary Table 1. List of the transcripts similarly deregulated in SOD1 ^G93A^** **motoneurons, at weeks 11 and 15 of age.**

| **Probe set ID** | **Symbol** | **Name** | **FC at 11w** | **FC at 15w** |
| --- | --- | --- | --- | --- |
| 1427351_s_at | Ighm | immunoglobulin heavy constant mu | 0.34 | 0.13 |
| 1423608_at | Itm2a | integral membrane protein 2A | 0.38 | 0.1 |
| 1438763_at | Dnahc2 | dynein, axonemal, heavy chain 2 | 0.39 | 0.4 |
| 1435815_at | Ldoc1 | leucine zipper, down-regulated in cancer 1 | 0.4 | 0.22 |
| 1423390_at | Siah1a | seven in absentia 1A | 0.41 | 0.36 |
| 1418916_a_at | Spp2 | secreted phosphoprotein 2 | 0.43 | 0.33 |
| 1451440_at | Chodl | chondrolectin | 0.46 | 0.13 |
| 1438213_at | A830018L16Rik | RIKEN cDNA A830018L16 gene | 0.52 | 0.4 |
| 1418072_at | Hist1h2bc | histone cluster 1, H2bc/ H2be/ H2bg | 0.56 | 0.48 |
| 1457248_x_at | Hsd17b7 | hydroxysteroid (17-beta) dehydrogenase 7 | 0.57 | 0.57 |
| 1441049_at | Kcna6 | Potassium voltage-gated channel, shaker-related, subfamily, member 6 | 0.59 | 0.46 |
| 1428427_at | Fbxl2 | F-box and leucine-rich repeat protein 2 | 0.6 | 0.6 |
| 1439006_x_at | Fam70a | family with sequence similarity 70, member A | 0.61 | 0.44 |
| 1456596_at | Fam70a | family with sequence similarity 70, member A | 0.61 | 0.34 |
| 1451660_a_at | Hoxb6 | homeobox B6 | 0.62 | 0.42 |
| 1441933_x_at | Pigz | phosphatidylinositol glycan anchor biosynthesis, class Z | 0.63 | 0.66 |
| 1460214_at | Pcp4 | Purkinje cell protein 4 | 0.63 | 0.68 |
| 1430977_at | Fam159b | family with sequence similarity 159, member B | 0.65 | 0.56 |
| 1417429_at | Fmo1 | flavin containing monooxygenase 1 | 0.66 | 0.68 |
| 1436948_a_at | Fam70a | family with sequence similarity 70, member A | 0.66 | 0.68 |
| 1426542_at | Endod1 | endonuclease domain containing 1 | 0.69 | 0.68 |
| 1455481_at | Ids | iduronate 2-sulfatase | 0.8 | 0.64 |
| 1449363_at | Atf3 | activating transcription factor 3 | 14.44 | 9.99 |
| 1449133_at | Sprr1a | small proline-rich protein 1A | 13.7 | 20.6 |
| 1434129_s_at | Lhfpl2 | lipoma HMGIC fusion partner-like 2 | 10.62 | 5.55 |
| 1454714_x_at | Phgdh | 3-phosphoglycerate dehydrogenase | 8.68 | 6.41 |
| 1437621_x_at | Phgdh | 3-phosphoglycerate dehydrogenase | 8.26 | 7.85 |
| 1426852_x_at | Nov | nephroblastoma overexpressed gene | 7.11 | 5.86 |
| 1422916_at | Fgf21 | fibroblast growth factor 21 | 6.09 | 2.67 |
| 1417022_at | Slc7a3 | solute carrier family 7 (cationic amino acid transporter, y+ system), member 3 | 5.14 | 2.14 |
| 1423427_at | Adcyap1 | adenylate cyclase activating polypeptide 1 | 4.91 | 5.62 |
| 1419665_a_at | Nupr1 | nuclear protein 1 | 4.52 | 3.1 |
| 1440142_s_at | Gfap | glial fibrillary acidic protein | 4.42 | 12.96 |
| 1426808_at | Lgals3 | lectin, galactose binding, soluble 3 | 4.22 | 8.85 |

**Supplementary Table 1. Continued.**

| **Probe set ID** | **Symbol** | **Name** | **FC at 11w** | **FC at 15w** |
| --- | --- | --- | --- | --- |
| 1426851_a_at | Nov | nephroblastoma overexpressed gene | 4.07 | 3.4 |
| 1447812_x_at | Flnc | filamin C, gamma | 3.41 | 3.23 |
| 1417516_at | Ddit3 | DNA-damage inducible transcript 3 | 3.35 | 3.73 |
| 1418778_at | Ccdc109b | coiled-coil domain containing 109B | 2.9 | 2.57 |
| 1417605_s_at | Camk1 | calcium/calmodulin-dependent protein kinase I | 2.63 | 2.35 |
| 1422302_s_at | Ftl1 | ferritin light chain 1 | 2.58 | 1.99 |
| 1455084_x_at | Shmt2 | serine hydroxymethyltransferase 2 (mitochondrial) | 2.58 | 2.47 |
| 1456471_x_at | Phgdh | 3-phosphoglycerate dehydrogenase | 2.58 | 4.14 |
| 1449319_at | Rspo1 | R-spondin homolog (Xenopus laevis) | 2.51 | 2.54 |
| 1448894_at | Akr1b8 | aldo-keto reductase family 1, member B8 | 2.48 | 2.35 |
| 1418364_a_at | Ftl1 | ferritin light chain 1 | 2.4 | 2.01 |
| 1450958_at | Tm4sf1 | transmembrane 4 superfamily member 1 | 2.36 | 2.53 |
| 1416431_at | Tubb6 | tubulin, beta 6 class V | 2.3 | 4 |
| 1448232_x_at | Tuba1a/b/c | tubulin, alpha 1A/1B/1C | 2.27 | 2.8 |
| 1451382_at | Chac1 | ChaC, cation transport regulator 1 | 2.27 | 1.78 |
| 1419666_x_at | Nupr1 | nuclear protein 1 | 2.21 | 2.03 |
| 1428942_at | Mt2 | metallothionein 2 | 2.14 | 2.43 |
| 1437832_x_at | Wars | tryptophanyl-tRNA synthetase | 2.11 | 1.44 |
| 1448135_at | Atf4 | activating transcription factor 4 | 2.08 | 1.45 |
| 1424635_at | Eef1a1 | eukaryotic translation elongation factor 1 alpha 1 | 2.07 | 1.73 |
| 1417491_at | Ctsb | cathepsin B | 2.01 | 2.36 |
| 1443883_at | Sys1 | SYS1 Golgi-localized integral membrane protein homolog | 2 | 2.02 |
| 1435893_at | Vldlr | very low density lipoprotein receptor | 1.96 | 2.3 |
| 1416128_at | Tuba1a/b/c | tubulin, alpha 1A/1B/1C | 1.94 | 2.26 |
| 1422507_at | Cstb | cystatin B | 1.9 | 1.93 |
| 1436094_at | Vgf | VGF nerve growth factor inducible | 1.9 | 1.8 |
| 1454607_s_at | Psat1 | phosphoserine aminotransferase 1 | 1.89 | 2.23 |
| 1438992_x_at | Atf4 | activating transcription factor 4 | 1.74 | 1.99 |
| 1434350_at | Csrnp1 | cysteine-serine-rich nuclear protein 1 | 1.72 | 2.46 |
| 1422506_a_at | Cstb | cystatin B | 1.67 | 1.99 |
| 1455908_a_at | Scpep1 | serine carboxypeptidase 1 | 1.64 | 1.76 |
| 1417103_at | Ddt | D-dopachrome tautomerase | 1.63 | 1.52 |
| 1424976_at | Rhov | ras homolog gene family, member V | 1.61 | 1.43 |
| 1422557_s_at | Mt1 | metallothionein 1 | 1.57 | 1.6 |
| 1420834_at | Vamp2 | vesicle-associated membrane protein 2 | 1.56 | 1.56 |
| 1454847_at | Lhfpl2 | lipoma HMGIC fusion partner-like 2 | 1.54 | 1.47 |

**Supplementary Table 1. Continued.**

| **Probe set ID** | **Symbol** | **Name** | **FC at 11w** | **FC at 15w** |
| --- | --- | --- | --- | --- |
| 1451596_a_at | Sphk1 | sphingosine kinase 1 | 1.53 | 1.54 |
| 1444010_at | Eif4e | eukaryotic translation initiation factor 4E | 1.51 | 2.11 |
| 1427918_a_at | Rhoq | ras homolog gene family, member Q | 1.49 | 1.39 |
| 1454764_s_at | Slc38a1 | solute carrier family 38, member 1 | 1.48 | 1.52 |
| 1426599_a_at | Slc2a1 | solute carrier family 2 (facilitated glucose transporter), member 1 | 1.48 | 1.53 |
| 1453111_a_at | Slc25a39 | solute carrier family 25, member 39 | 1.48 | 1.39 |
| 1437711_x_at | Odc1 | ornithine decarboxylase, structural 1 | 1.41 | 1.45 |
| 1435902_at | Nudt18 | nudix (nucleoside diphosphate linked moiety X)-type motif 18 | 1.4 | 1.6 |
| 1450421_at | Tgfa | transforming growth factor alpha | 1.39 | 1.52 |
| 1434773_a_at | Slc2a1 | solute carrier family 2 (facilitated glucose transporter), member 1 | 1.38 | 1.74 |
| 1424831_at | Cpne2 | copine II | 1.37 | 1.38 |
| 1434784_s_at | Tmem106c | transmembrane protein 106C | 1.36 | 1.5 |
| 1416290_a_at | Psmc4 | proteasome (prosome, macropain) 26S subunit, ATPase 4 | 1.3 | 1.45 |
